# Association between lung function decline and obstructive sleep apnoea: the ALEC study

**DOI:** 10.1007/s11325-020-02086-1

**Published:** 2020-07-06

**Authors:** Össur Ingi Emilsson, Fredrik Sundbom, Mirjam Ljunggren, Bryndis Benediktsdottir, Judith Garcia-Aymerich, Dinh Son Bui, Deborah Jarvis, Anna-Carin Olin, Karl A. Franklin, Pascal Demoly, Eva Lindberg, Christer Janson, Thor Aspelund, Thorarinn Gislason

**Affiliations:** 1 Department of Respiratory, Allergy and Sleep Research, Akademiska Sjukhuset, Uppsala University, 751 85 Uppsala, Sweden; 2 Department of Respiratory Medicine and Allergology, Akademiska Sjukhuset, Uppsala, Sweden; 3 Department of Sleep Medicine, Landspitali, Reykjavik, Iceland; 4 Faculty of Medicine, University of Iceland, Reykjavik, Iceland; 5 ISGlobal, Barcelona, Spain; 6 Universitat Pompeu Fabra (UPF), Barcelona, Spain; 7 CIBER Epidemiología y Salud Pública (CIBERESP), Barcelona, Spain; 8 Allergy and Lung Health Unit, Centre for Epidemiology and Biostatistics, University of Melbourne, Melbourne, Australia; 9 National Heart and Lung Institute, Imperial College London, London, UK; 10 Unit of Occupational and Environmental Medicine, University of Gothenburg, Gothenburg, Sweden; 11 Department of Surgical and Perioperative Sciences, Umeå University, Umeå, Sweden; 12 Department of Pulmonology, Division of Allergy, Hôpital Arnaud de Villeneuve, University Hospital of Montpellier, Montpellier, France; 13 Inserm, Sorbonne Université, Equipe EPAR – IPLESP, Paris, France; 14 Centre for Public Health Sciences, University of Iceland, Reykjavik, Iceland

**Keywords:** Sleep apnoea, Lung function, Lung function decline, Asthma

## Abstract

**Purpose:**

To study changes in lung function among individuals with a risk of obstructive sleep apnoea (OSA), and if asthma affected this relationship.

**Methods:**

We used data from the European Community Respiratory Health Survey II and III, a multicentre general population study. Participants answered questionnaires and performed spirometry at baseline and 10-year follow-up (*n* = 4,329 attended both visits). Subjects with high risk for OSA were identified from the multivariable apnoea prediction (MAP) index, calculated from BMI, age, gender, and OSA symptoms at follow-up. Asthma was defined as having doctor’s diagnosed asthma at follow-up. Primary outcomes were changes in forced expiratory volume in 1 s (FEV1) and forced vital capacity (FVC) from baseline to follow-up.

**Results:**

Among 5108 participants at follow-up, 991 (19%) had a high risk of OSA based on the MAP index. Participants with high OSA risk more often had wheeze, cough, chest tightness, and breathlessness at follow-up than those with low OSA risk. Lung function declined more rapidly in subjects with high OSA risk (low vs high OSA risk [mean ± SD]: FEV1 = − 41.3 ± 24.3 ml/year vs − 50.8 ± 30.1 ml/year; FVC = − 30.5 ± 31.2 ml/year vs − 45.2 ± 36.3 ml/year). Lung function decline was primarily associated with higher BMI and OSA symptoms. OSA symptoms had a stronger association with lung function decline among asthmatics, compared to non-asthmatics.

**Conclusion:**

In the general population, a high probability of obstructive sleep apnoea was related to faster lung function decline in the previous decade. This was driven by a higher BMI and more OSA symptoms among these subjects. The association between OSA symptoms and lung function decline was stronger among asthmatics.

**Electronic supplementary material:**

The online version of this article (10.1007/s11325-020-02086-1) contains supplementary material, which is available to authorized users.

*Study rationale:* Data on lung function changes in obstructive sleep apnoea (OSA) patients is lacking. Co-morbid asthma might affect such an association. This multi-centre general population study with 10 years of follow-up (*n* = 4329) sought an association between the high risk of OSA and lung function decline.

*Study impact:* A clinical OSA risk score identified participants that declined more rapidly in lung function than others. This effect seemed more pronounced among asthmatics. This finding is potentially clinically relevant both for OSA patients and for asthma patients with comorbid OSA but needs further prospective validation.

## Introduction

Sleep-disordered breathing, ranging from habitual snoring to obstructive sleep apnoea (OSA), is increasingly being recognised as a common problem in the general population [1]. When OSA is accompanied by daytime sleepiness, the estimated prevalence of 6% among males and 4% among females has been reported, but the numbers are higher when only based on the number of breathing cessations during sleep [1, 2]. Between 7 and 23% in the adult population snore habitually [3, 4]. Habitual snoring and breathing cessations commonly co-occur but can also appear separately and do not necessarily have the same clinical implications [5–8].

Habitual snoring has recently been described to be associated with a decline in lung function, indicating that OSA may play a role in lung function decline [9]. However, even though snoring and OSA are linked, not all habitual snorers have OSA [5]. The precise roles of snoring, on the one hand, and apnoeas, on the other, are therefore unknown in relation to lung function decline.

OSA is associated with respiratory symptoms and asthma, even though the nature or directionality of the association is not established [10–13]. The combination of OSA and asthma may be detrimental. For example, subjects with the combination of OSA and asthma have a lower oxygen saturation during night than subjects with either OSA or asthma alone [14]. Additionally, a small retrospective study found an association between OSA and lung function decline among asthmatics [15]. Otherwise, the long-term effects of OSA on lung function are unknown [10, 11].

A whole-night sleep study is needed to diagnose OSA. However, sleep studies are difficult to perform in large scale. The multivariable apnoea prediction (MAP) index is a validated clinical instrument to identify subjects with a high risk of OSA [16, 17]. It is simple to apply and gives a score between 0 and 1, where a score above 0.5 is considered a high risk of OSA. A score above 0.5 is reported to have a sensitivity of 88% and a specificity of 55% in a sleep centre patient population [16].

Our aim was to study in a long-term follow-up study of a large, general population–based, middle-aged cohort whether a high risk of OSA, assessed by the MAP index, is associated with a faster lung function decline and, if so, if a diagnosis of asthma affects such an association.

## Methods

### Study cohort

This study is a part of the larger Ageing Lungs in European Cohorts (ALEC) study (www.alecstudy.org), which is a research collaboration aimed to improve the knowledge on risk factors for lung diseases and lung function decline. For the current study, we used material from the European Community Respiratory Health Survey (ECRHS) II and ECRHS III, performed in 2000 and 2010. The ECRHS is a prospective, international, population-based cohort study [18, 19]. In total, 5833 participated in ECRHS III. Data for calculating MAP was available on 5108 (88%) participants from 22 European centres and one centre in Melbourne, Australia. Thereof, 4329 (85%) participants with a MAP index had also attended ECRHS II.

Participants answered the same questions on respiratory symptoms, diagnoses and comorbidities at both baseline and follow-up. They also performed a spirometry on both visits, but reversibility testing was only performed in ECRHS III. Data for the MAP index was only available in ECRHS III. A sleep study was performed only in Iceland [2]. From the sleep studies, we calculated the apnoea-hypopnoea index (AHI) and the oxygen desaturation index (ODI) (see the Appendix).

Due to a significant dropout of participants over the 20-year study period from ECRHS I to ECRHS III, the possibility of a selection bias has been studied previously. Slight differences were found between long-term participants and participants lost to follow-up, but generally of minimal importance [20].

### MAP index

The MAP index identifies subjects who are at risk of OSA [16, 17]. In short, the index is calculated based on four factors: age, gender, BMI and a composite score from reported symptoms such as loud snoring, apnoeas and choking during sleep (hereafter ‘OSA symptom score’, described below). The final calculated MAP index has a range from 0 to 1.

The range of the OSA symptom score is between 0 and 4. It is calculated as an average score from three questions: “Have you been told that you stop breathing or have irregular breathing while you are sleeping?”; “Have you been woken up all of a sudden with a choking sensation or not being able to breathe?”; “Have you been told that you snore loudly or that your snoring disturbs other people?” The participants reported if they had these symptoms on average in the past 12 months: ‘Never’, ‘Seldom’, ‘Sometimes’, ‘Frequently’ and ‘Every time’. A higher symptom score associates with a higher prevalence of OSA, even though no cut-off value has been studied for sensitivity and specificity of OSA [16].

Data for calculating the MAP index was only available at ECRHS III. Participants with a MAP index of 0.5 or higher were classed as ‘high OSA risk’, while participants with a score below 0.5 were classed as ‘low OSA risk’ [16].

### Asthma

Participants were defined as having asthma if they reported having a doctor’s diagnosed asthma at follow-up [21]. For consistency, asthma was defined at ECRHS III, similar to the MAP index. Current asthma was defined as currently using asthma medication and/or reported asthma exacerbation in the previous 12 months, in addition to having a doctor’s diagnosed asthma.

### Spirometry

Lung function was measured similarly by spirometry on both visits [22]. All subjects made at least five forced expiratory manoeuvres, and maximal values with at least 150 ml reproducibility were used for analysis. Predicted values were calculated using reference equations from the Global Lung Function Initiative (GLI) [23].

In ECRHS III only, forced expiratory volume in 1 s (FEV_1_) and forced vital capacity (FVC) were also recorded post bronchodilation with 200 μg salbutamol.

Change in lung function from ECRHS II to ECRHS III was calculated both as change in % of predicted between the surveys, as well as ml/year using the actual time between the two spirometries for calculation.

### Statistical analysis

The statistical program Stata (version 14.2; Stata Inc., USA) was used for statistical analysis. For cross-sectional analysis of MAP index associations with symptoms and lung function, chi-square test, linear regression and *t* test were used as appropriate. Before performing linear regression and *t* test, data was checked for normal distribution.

The unadjusted analysis of change in lung function was performed with *t* test. The adjusted analysis was performed using a mixed linear regression model, with centre as a random effect. The primary effect variable was the MAP index, and the outcome variable was change in lung function parameters between visits, while adjusting for baseline value.

We evaluated the effects of each factor of the MAP index, using a single mixed regression model to evaluate all factors simultaneously. We evaluated the association pattern between the OSA symptom score and changes in lung function and found it to be linear, and therefore analysed the OSA symptom score as a continuous variable (see Appendix, Figures A2 and A3). The results were then stratified by asthma status.

As a sensitivity analysis, three modifications of the analysis were performed. First, some calculations were rerun only on those with a change in BMI of no more than 2 kg/m^2^ between study visits, as increasing BMI can significantly affect lung function and would likely have significantly changed the MAP index score between visits [24]. Second, we exchanged ‘asthma’ for ‘current asthma’ in calculations on lung function decline. Third, we added AHI or ODI from sleep studies to the mixed regression models on MAP index factors and lung function decline (Appendix).

## Results

### Population characteristics

Among the 5108 participants, 991 (19.4%) were classified as having a high risk of OSA. Participants with high OSA risk were older, heavier and more often male, as expected (Table 1). They also more often had a history of smoking, although current smoking status did not differ. Doctor’s diagnosed asthma had a prevalence of 17%. Participants with a high risk of OSA more often reported respiratory symptoms, i.e. wheezing, breathlessness, cough and phlegm (Table 1).

**Table 1:** Population characteristics at ECRHS III

	Low OSA risk (*n* = 4117)	High OSA risk (*n* = 991)
Age, mean ± SD	53.4 ± 7.0	57.2 ± 6.4
Male gender, %	39	87
BMI, median (IQR)	25.5 (23.2–28.1)	31.2 (28.8–35.0)
Doctor’s diagnosed asthma, %	18	17
Smoking status		
Never smoker, %	44	30
Former smoker, %	39	52
Current smoker, %	17	18
OSA symptoms		
Apnoeas (at least ‘sometimes’), %	8	42
Choking (at least ‘sometimes’), %	4	16
Snoring (at least ‘frequently’), %	10	43
Respiratory symptoms		
Wheeze, %	22	35
Nocturnal chest tightness, %	13	19
Breathlessness at rest, %	7	9
Breathlessness after effort, %	20	32
Nocturnal breathlessness, %	7	15
Woken by cough, %	32	33
Woken by cough ≥ 1/month, %	12	19
Morning cough, %	10	18
Morning phlegm, %	12	21

Data for participants in both ECRHS II and ECRHS III (*n* = 4329) was not significantly different (data not shown)

#### Validity of the MAP index

In the Icelandic population that underwent a whole-night sleep study [2], the sensitivity and specificity of the MAP index > 0.5 to identify moderate sleep apnoea, i.e. an AHI of > 15, were 58% and 84%, respectively (see the Appendix).

### Cross-sectional lung function

Participants with high OSA risk had a significantly lower lung function at follow-up (Table 2). The spirometry showed lower lung volumes among those with a high risk of OSA, but also slightly more obstruction among males. Reversibility of FEV_1_ was similar in both groups.

**Table 2:** Lung function data at ECRHS III (unadjusted), gender-stratified because of the unequal gender distribution between the study groups (low OSA risk: *n* = 4117; high OSA risk: *n* = 991)

	Males			Females		
	Spirometry data (mean ± SD)		*p* value	Spirometry data (mean ± SD)		*p* value
	Low OSA risk (*n* = 1592)	High OSA risk (*n* = 859)		Low OSA risk (*n* = 2525)	High OSA risk (*n* = 132)	
FEV_1_ pre (L)	3.66 ± 0.67	*3.28 ± 0.65*	*< 0.001*	2.58 ± 0.49	*2.29 ± 0.46*	*< 0.001*
FEV_1_ pre (% pred)	95.8 ± 14.3	*91.1 ± 15.7*	*< 0.001*	95.4 ± 14.5	*89.5 ± 13.7*	*< 0.001*
FVC pre (L)	4.86 ± 0.81	*4.38 ± 0.77*	*< 0.001*	3.40 ± 0.58	*2.97 ± 0.56*	*< 0.001*
FVC pre (% pred)	99.9 ± 13.0	*94.7 ± 13.6*	*< 0.001*	100.1 ± 13.4	*92.2 ± 12.3*	*< 0.001*
FEV_1_/FVC pre (%)	75.4 ± 6.6	*74.8 ± 6.9*	*0.04*	75.9 ± 6.5	77.0 ± 5.8	0.05
Reversibility of FEV_1_ (%)	2.57 ± 4.65	2.72 ± 5.13	0.50	2.95 ± 5.27	2.83 ± 5.72	0.81

Statistically significant differences (*p* < 0.05) indicated by italics

### Longitudinal lung function

#### OSA risk and lung function decline

High OSA risk at follow-up was associated with a faster decline in lung function over the previous decade (Table 3). After adjusting for pack-years, centre and baseline value, a high OSA risk was still significantly associated with a faster decline in lung function. The difference between the groups in lung function decline was more pronounced for FVC than for FEV_1_. The decrease in FEV_1_/FVC was consequently smaller among those with a high OSA risk. When stratified by asthma, the faster decline in participants with high OSA risk was found in both subjects with and without asthma (Table 4).

**Table 3:** Lung function decline between ECRHS II and ECRHS III by OSA risk (MAP index > 0.5)

	Spirometry data (mean ± SD)		*p* value
	Low OSA risk (*n* = 3475)	High OSA risk (*n* = 854)	
Between visits 2 and 3			
Change in FEV_1_ (ml/year)	− 41.3 ± 24.3	*− 50.8 ± 30.1*	*< 0.001*
Difference in % predicted FEV_1_*	− 4.50 ± 8.38	*− 5.80 ± 9.50*	*< 0.001*
Change in FVC (ml/year)	− 30.5 ± 31.2	*− 45.2 ± 36.3*	*< 0.001*
Difference in % predicted FVC*	− 0.70 ± 8.71	*− 3.01 ± 9.04*	*< 0.001*
Difference in FEV_1_/FVC*	− 4.93 ± 4.68	*− 4.24 ± 4.66*	*< 0.001*

Statistically significant differences (*p* < 0.05) indicated by italics

*Difference in percentage points

**Table 4:** Difference between high and low OSA risks in lung function changes

	Spirometry data, coefficient (95% CI)	
	High vs low OSA risk (unadjusted)	High vs low OSA risk (adjusted)
Without asthma (*n* = 4202)		
Change in FEV_1_ (ml/year)	*− 10.4 (− 12.5; − 8.2)*	*− 5.0 (− 7.1; − 2.9)*
Difference in % predicted FEV_1_*	*− 1.46 (− 2.18; − 0.74)*	− 0.40 (− 1.12; 0.33)
Change in FVC (ml/year)	*− 15.6 (− 18.4; − 12.7)*	*− 11.20 (− 14.1; − 8.3)*
Difference in % predicted FVC*	*− 2.46 (− 3.23; − 1.69)*	*− 1.67 (− 2.45; − 0.89)*
Difference in FEV_1_/FVC*	*0.56 (0.16; 0.97)*	*0.83 (0.43; 1.23)*
With asthma (*n* = 894)		
Change in FEV_1_ (ml/year)	− 4.9 (− 10.5; 0.7)	− 2.11 (− 7.6; 3.3)
Difference in % predicted FEV_1_*	− 0.54 (− 2.53; 1.45)	0.09 (− 1.96; 2.13)
Change in FVC (ml/year)	*− 10.7 (− 17.3; − 4.1)*	*− 7.6 (− 14.2; − 1.0)*
Difference in % predicted FVC*	− 1.70 (− 3.59; 0.19)	− 0.94 (− 2.84; 0.96)
Difference in FEV_1_/FVC*	*1.38 (0.27; 2.49)*	*1.40 (0.30; 2.50)*

Unadjusted values and values adjusted for pack-years, centre and baseline spirometry value. Separate calculations by asthma status are presented

Statistically significant differences (*p* < 0.05) indicated by italics

*Difference in percentage points

#### MAP index factors, asthma and lung function decline

**Fig. 1:**
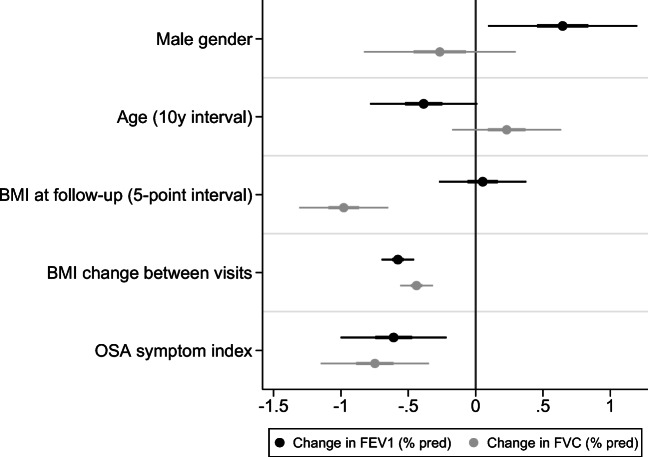
Multivariate mixed regression model on change in percent predicted of FEV_1_ and FVC between visits, showing coefficients with 50% and 95% confidence intervals. Adjusted for pack-years, baseline spirometry value and centre. Note that variables differ in scale (*categorical*, gender; *ordinal*, OSA symptom score; *continuous*, BMI, age), affecting the interpretation of each coefficient. OSA, obstructive sleep apnoea; % pred, percent predicted

To further analyse which elements of the OSA risk score (MAP index) that were most important in these associations, we replaced the MAP-based OSA risk categories in the model for its specific factors, that is age, gender, BMI and symptom index. We found that after adjusting for confounding factors, a higher OSA symptom score (based on nocturnal snoring, apnoea and a choking sensation) and an increase in BMI independently associated with a more rapid decline in percent predicted FEV_1_ and FVC over time (Fig. 1). Additionally, a higher BMI at follow-up associated with a more rapid decline in percent predicted FVC, but not in FEV_1_.

**Fig. 2:**
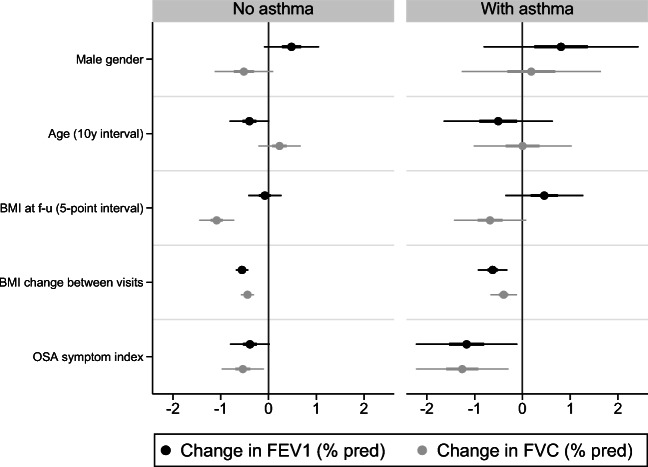
Multivariate mixed regression model on change in percent predicted of FEV_1_ and FVC between visits, showing coefficients with 50% and 95% confidence intervals. Adjusted for pack-years, baseline spirometry value and centre. Subgraphs by doctor’s diagnosed asthma. Note that variables differ in scale (*categorical*, gender; *ordinal*, OSA symptom score; *continuous*, BMI, age), affecting the interpretation of each coefficient. OSA, obstructive sleep apnoea; f-u, follow-up; % pred, percent predicted

When the results were stratified by asthma, we found that the association between the OSA symptom score and decline in percent predicted FEV_1_ was only significant among those with asthma (Fig. 2). We also found the association between the OSA symptom score and decline in percent predicted FVC to be somewhat more pronounced among those with asthma (Fig. 2). An interaction analysis did, however, not find a statistically significant interaction between asthma and the OSA symptom score (*p* = 0.24 and *p* = 0.35 for FEV_1_ and FVC, respectively).

Because BMI is a significant confounder in the association between OSA symptoms and lung function changes, we performed further analysis to minimise the confounding effects of large changes in weight. We redid the analysis using only those with a relatively unchanged BMI between visits (change in BMI less than 2 kg/m^2^, *n* = 2247). This group had a mean BMI at follow-up of 25.6 kg/m^2^ (without vs with asthma (mean ± SD): 25.6 ± 3.8 kg/m^2^ vs 25.7 ± 4.0 kg/m^2^). The results showed only those with asthma had a significant association between the OSA symptom score and decline in lung function (see the Appendix, Figure A4), and an additive interaction term between the OSA symptom score and asthma (*p* = 0.01 and *p* = 0.09 for FEV_1_ and FVC, respectively).

##### Sensitivity analysis

A sensitivity analysis looking at current asthma did not change the pattern of the results on changes in lung function in relation to the MAP factors, although the association between the OSA symptom score and lung function became non-significant.

Using data from the Icelandic sleep study subgroup, we added ODI and AHI to the model above. The associations between an elevated OSA symptom score and a decline in FEV_1_ and FVC remained significant, only among asthmatics. The ODI and AHI were not independently associated with lung function decline (see the Appendix, Tables A1a, A1b, A2a and A2b).

## Discussion

In this study, we found that having a high risk of obstructive sleep apnoea (OSA), as measured by the minimal apnoea prediction (MAP) index, was associated with more respiratory symptoms and poorer spirometry results. We also found that a high OSA risk associated with a more rapid decline in lung function in the past 10 years. This effect was mostly driven by BMI and OSA symptoms, which constitute a part of the OSA risk score (MAP index). When stratified by asthma, we found that the effect of OSA symptoms on lung function decline was more prominent among asthmatics.

### Lung function decline

A high risk of OSA, defined by the MAP index, was associated with a generally more restrictive spirometry pattern with significantly lower FEV_1_ and FVC but a similar FEV_1_/FVC ratio. This may, in part, reflect effects of higher BMI. Nonetheless, a study with BMI-matched participants did also find a significant association between more severe OSA and a lower lung function [25]. We also found that lung function had declined more rapidly in the previous 10 years among those with a positive MAP index. This effect on lung function was more prominent among non-asthmatics.

BMI is an important confounder in the association between OSA risk and lung function, but as BMI constitutes a big part of the MAP index, a simple adjustment for BMI was not statistically appropriate. Instead, we further analysed which factors composing the MAP index (gender, age, BMI and OSA symptom score) were most important for the found changes in lung function. An increase in BMI over the study period was associated with a steeper decline in FEV_1_ and FVC and a higher BMI at follow-up associated with a steeper decline in FVC, independent of asthma status. A similar decline in lung function with increasing BMI has been described previously [24]. Independent of the BMI effects, we also found the OSA symptom score to associate with a steeper decline in FEV_1_ and FVC.

We have recently described that snoring is associated with a significant decline in lung function [9], and this study further validates that finding by also looking at reported nocturnal choking and apnoeas, other common OSA symptoms. We even found a negative linear relationship between the OSA symptom score and the decline in lung function, further supporting the relevance of this finding. Importantly, this effect was not modified by adding apnoea-hypopnoea index (AHI) to the model in our subset of participants with sleep studies, suggesting that the symptoms themselves are more important in this association than the classic AHI measurement.

Snoring and apnoeas are both associated with an increased respiratory effort during sleep [26]. Vibrations caused by snoring may have detrimental effects on structures in the proximity of the upper airways. For example, heavy snorers have more carotid artery stenosis than mild snorers, irrespective of their AHI, but with no differences in their femoral arteries [6, 27]. We hypothesise symptoms of OSA may also cause damage to the airways through similar mechanisms of vibration and increased respiratory effort. However, whether the decline in lung function precedes the development of OSA is also debatable. The role of systemic inflammation and hypoxia has also been proposed but could not be assessed in the current study [28]. More prospective studies are therefore needed to better study the causal association between OSA and lung function decline.

Importantly, the association between the OSA symptom score and lung function decline was not explained by weight changes and was more pronounced among those with comorbid asthma. Among participants with stable weight over the study period, the association between the OSA symptom score and lung function decline was only significant among asthmatics. This contrasts our finding of a decreased association between the MAP index and lung function decline among asthmatics. A likely explanation for this discrepancy is that other factors constituting the MAP index, such as BMI, had a lesser effect on lung function decline among asthmatics, even though BMI at follow-up and change in BMI between visits was similar between asthmatics and non-asthmatics. Therefore, the net effect of the MAP index was less among asthmatics. This complex interplay between OSA symptoms, asthma and lung function needs to be studied further.

### Implications

The association between OSA and respiratory symptoms is well established [12, 29]. This study adds evidence of an association between elevated OSA risk and a decline in lung function in the previous 10 years, and that the MAP index may be useful to identify these subjects. Two factors were most important in this context: BMI and OSA symptoms. When adding classic OSA parameters from a subgroup undergoing sleep study (AHI and ODI), the model was not improved, indicating that these measurements may not fully capture the negative respiratory effects of sleep-disordered breathing. Indeed, numerous studies have shown the AHI (a usual OSA diagnostic index) does not encompass the diversity of OSA [7, 8, 30, 31]. For example, one cluster analysis study found that certain OSA phenotypes were a stronger predictor of adverse cardiovascular events than the AHI itself [32]. Our finding of an association between OSA symptoms and lung function changes, irrespective of AHI, indicates that better markers of OSA need to be sought and further support the importance of different clinical OSA phenotypes.

The association between OSA symptoms and lung function decline was somewhat more prominent among asthmatics. This supports current asthma guidelines that recommend screening for OSA in selected patients.

### Strengths and limitations

The main strengths of this study are the large, well-defined, general population, with high-quality spirometry and a 10-year follow-up time, and a subgroup undergoing a whole-night sleep study. However, this study also had some weaknesses. The major limitation was we could not calculate the MAP index at baseline, and we were therefore bound to study changes in lung function prior to the MAP index calculation. This weakens any conclusions about causality, and we cannot exclude that the cause and effect relationship could go in the opposite direction (i.e. a more rapid decline in lung function increasing the risk of a positive MAP index). Second, we did not have data to analyse asthma severity in detail, which may be a confounder for lung function decline among asthmatics. However, we performed an analysis adjusting for asthma exacerbations in the previous year as a surrogate marker and found that this did not change the pattern of the results (data not shown). Third, we only had participants from one centre undergoing objective sleep measurements and therefore had to use a surrogate measure for OSA. Our surrogate measure, MAP index, identified in a subcohort roughly 58% of participants with an AHI above 15; however, 57% of those with a positive MAP index had an AHI below 15. Therefore, many of those with a positive MAP index in our cohort may not have OSA. On the other hand, they have other signs of sleep-disordered breathing such as snoring and choking, which may be detrimental by itself. As we found a stronger association between these symptoms and lung function decline among subjects with asthma, these symptoms may be more associated to an asthma phenotype with a more rapid lung function decline. We also do not know if, in the OSA risk group, there is a difference in lung function outcome between those who ultimately develop OSA and those who do not.

## Conclusion

In this large general population–based multicentre study, we found that participants with a high probability of OSA also had an increased prevalence of respiratory symptoms. A high risk of OSA was related to faster lung function decline in the previous decade, and this relationship was more marked in people with asthma.

## Appendix

ESM 1 (PDF 373 kb)

## Abbreviations

AHI: Apnoea-hypopnoea index
ALEC: Ageing Lungs in European Cohorts
ECRHS: European Community Respiratory Health Survey
FEV_1_: Forced expiratory volume in 1 s
FVC: Forced vital capacity
MAP: Multivariable apnoea prediction
OSA: Obstructive sleep apnoea
ODI: Oxygen desaturation index
