# Supplementary material for: Association between lung function decline and obstructive sleep apnoea: the ALEC study
Source: Sleep Breath. 2020 Jul 6;25(2):587–96. doi: 10.1007/s11325-020-02086-1 (PMC8195766; doi:10.1007/s11325-020-02086-1)
Supplement: ESM 1 — (PDF 373 kb) [file 11325_2020_2086_MOESM1_ESM.pdf]

Appendix – Sleep measurements and spirometry

Association between lung function decline and obstructive sleep apnea: The ALEC study

Össur Ingi Emilsson, Fredrik Sundbom, Mirjam Ljunggren, Bryndis Benediktsdottir, Judith Garcia-Aymerich, Dinh Son Bui, Deborah Jarvis, Anna-Carin Olin, Karl A. Franklin, Pascal Demoly, Eva Lindberg, Christer Janson, Thor Aspelund, Thorarinn Gislason

Sleep study - Icelandic subcohort

Among the 455 Icelandic participants in ECRSH III, altogether 416 underwent a whole night sleep study, with a T3 device (Nox Medical, Reykjavik, Iceland). A detailed description of the measurements has been published.[2] In short, two trained sleep technologists scored the sleep studies. Studies had to have ≥4 h of scorable oxygen saturation and more than two out of three respiratory traces: cannula flow, thorax and respiratory inductive plethysmography belts. Hypopneas were classified as a ≥30% drop in respiratory flow for ≥10 s with ≥4% oxygen desaturation.

Complete sleep study data was available on 401 participants. Thereof, 334 participants had data for MAP index calculation. Additionally, 9 participants with data for MAP index (5 with a MAP index > 0.5) had ongoing treatment for obstructive sleep apnea, and did therefore not undergo a sleep study. We included these participants in the group of participants with AHI > 15.

According to the Icelandic general population data, the sensitivity of the MAP index is 34/59=58%, and the specificity is 239/284=84% (figure A1).

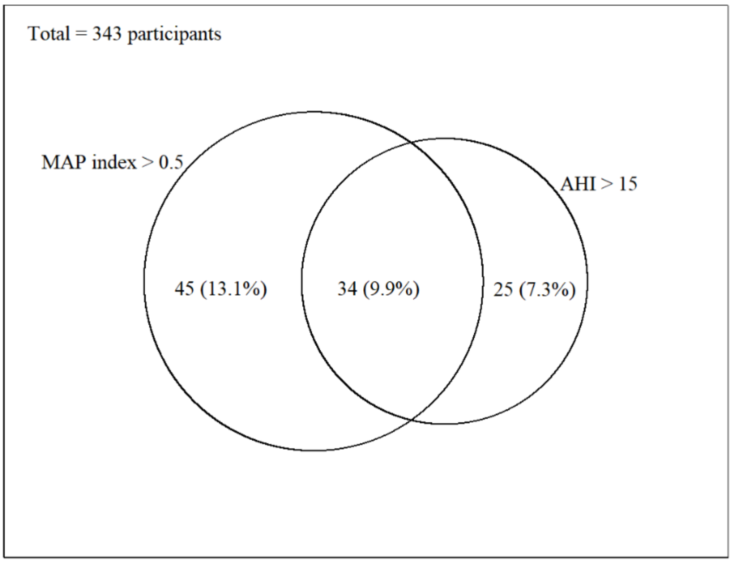

Figure A1. Venn diagram of the relationship between a positive MAP index and AHI (including those with previously diagnosed OSA as “AHI > 15”).

AHI, ODI, OSA symptom index, and change in spirometry

Icelandic participants with sleep study in ECRSH III and spirometry at baseline and follow-up were 295. Thereof, 277 had answered the OSA symptom index questions.

Table A1a. Association of **ODI** and OSA symptom index with change in **percent predicted FEV1** over 10 years. Adjusted for age, gender, BMI at follow-up, BMI change between visits, pack-years and baseline FEV1 value (n=277).

Coef. (95% CI)	No asthma (N = 197)	With asthma (N = 59)
ODI (sqrt-transf)	-0.19 (-1.05; 0.66)	3.00 (-0.06; 6.06)
OSA symptom score	-0.77 (-2.44; 0.90)	**-8.64 (-13.68; -3.61)**

Table A1b. Association of **ODI** and OSA symptom index with change in **percent predicted FVC** over 10 years. Adjusted for age, gender, BMI at follow-up, BMI change between visits, pack-years and baseline FVC value (n=277).

Coef. (95% CI)	No asthma (N = 198)	With asthma (N = 58)
ODI (sqrt-transf)	-0.18 (-0.99; 0.63)	1.38 (-1.09; 3.85)
OSA symptom score	-0.31 (-1.88; 1.26)	**-6.79 (-10.90; -2.69)**

Table A2a. Association of **AHI** and OSA symptom index with change in **percent predicted FEV1** over 10 years. Adjusted for age, gender, BMI at follow-up, BMI change between visits, pack-years and baseline FEV1 value (n=277).

Coef. (95% CI)	No asthma (N = 189)	With asthma (N = 58)
AHI (ln-transf)	-0.42 (-1.27; 0.42)	0.83 (-1.70; 3.37)
OSA symptom score	-0.72 (-2.42; 0.98)	-**6.86 (-11.82; -1.90)**

Table A2b. Association of **AHI** and OSA symptom index with change in **percent predicted FVC** over 10 years. Adjusted for age, gender, BMI at follow-up, BMI change between visits, pack-years and baseline FVC value (n=277).

Coef. (95% CI)	No asthma (N = 189)	With asthma (N = 57)
AHI (ln-transf)	-0.37 (-1.17; 0.43)	0.31 (-1.65; 2.27)
OSA symptom score	-0.28 (-1.88; 1.32)	**-5.86 (-9.71; -2.02)**

OSA symptom index and change in lung function

The obstructive sleep apnea (OSA) symptom index had a linear relationship to changes in FEV1 and FVC (figures A2 and A3) (n=3419).

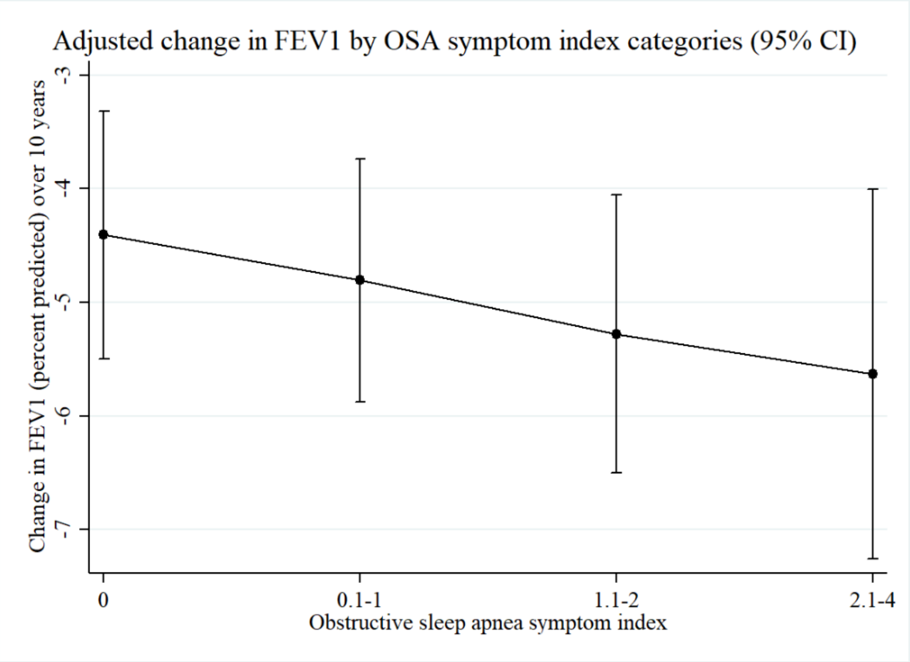

Figure A2. Change in FEV1 between study visits, by obstructive sleep apnea symptom index. Adjusted for age, gender, BMI, smoking, centre, and baseline FEV1 value.

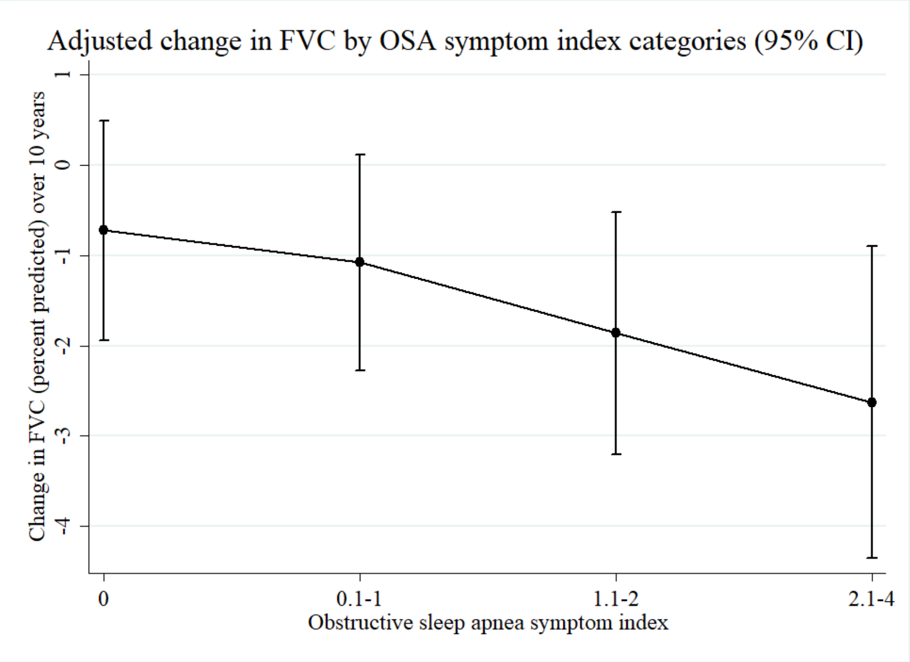

Figure A3. Change in FVC between study visits, by obstructive sleep apnea symptom index. Adjusted for age, gender, BMI, smoking, centre, and baseline FVC value.

BMI adjustments – sensitivity analysis

As changes in BMI had a significant effect on changes in lung function, we redid the analysis by only analyzing those with a change in BMI of more or less than 2 kg/m^2 (figure A5, n=1940). The association of high OSA risk to difference in percent predicted FEV1 became non-significant, while the association to difference in percent predicted FVC remained significant (Table A3).

The association of the OSA symptom index to changes in FEV1 and FVC became non-significant among those without asthma, but remained significant among those with asthma (Figure A4), and a significant interaction was found between the OSA symptom score and asthma.

Table A3. Lung function decline between ECRHS II and III by OSA risk (MAP index >0.5), among participants with change in BMI ± 2 kg/m^2 between visits.

**Spirometry data (mean ± SD)**	Low OSA risk (n = 1873)	High OSA risk (n = 327)	p-value
**Between visits 2 and 3**			
Change in FEV1 (ml/year)	-40.2 ± 23.3	**-46.6 ± 28.1**	**<0.001**
Difference in % predicted FEV1*	-4.14 ± 8.13	-4.62 ± 8.65	0.34
Change in FVC (ml/year)	-28.9 ± 30.5	**-39.7 ± 32.8**	**<0.001**
Difference in % predicted FVC*	-0.32 ± 8.54	**-1.86 ± 8.22**	**0.004**
Difference in FEV1/FVC*	-4.86 ± 4.74	**-4.12 ± 4.23**	**0.01**

**Difference in percentage points*

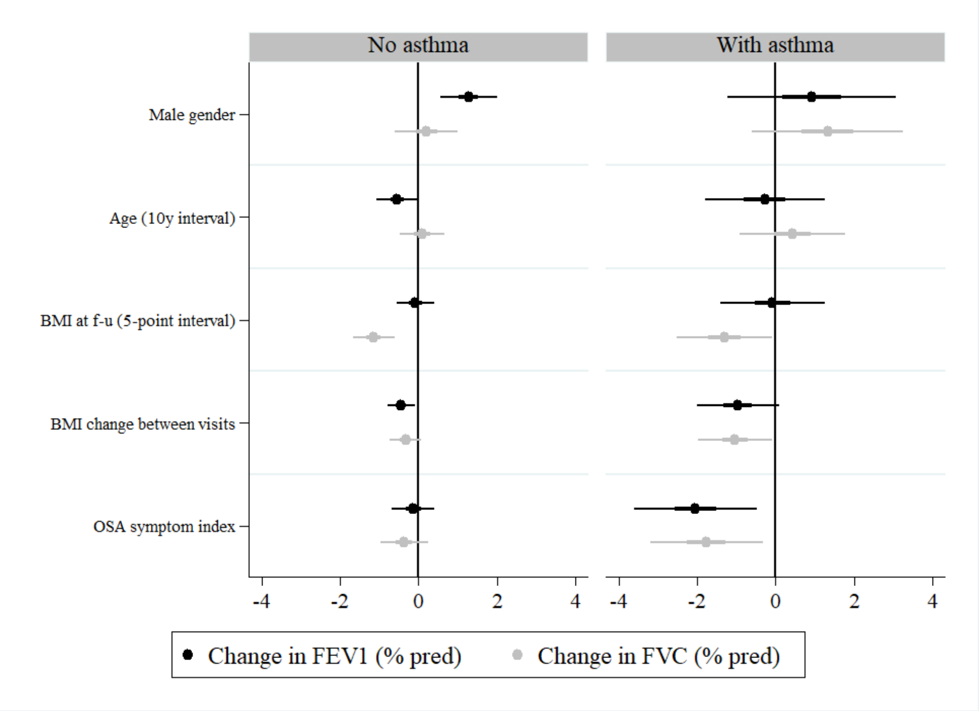

Figure A4. Multivariate mixed regression model on change in percent predicted of FEV1 and FVC between visits, only among participants with a change in BMI of less than ± 2 kg/m^2. The graph shows regression coefficients with 50 and 95% confidence intervals. Adjusted for pack-years, baseline spirometry value, and centre. Subgraphs by doctor's diagnosed asthma. Note that variables differ in scale (*categorical:* gender; *ordinal:* OSA symptom index; *continuous:* BMI, age), affecting the interpretation of each coefficient. OSA: Obstructive sleep apnea; f-u: follow-up; % pred: percent predicted.

Current asthma – sensitivity analysis

Analysis by "current asthma" (compared to "doctor's diagnosed asthma" as in the main article) did not change the pattern of the results on changes in lung function in relation to the MAP factors, albeit some indices became non-significant (Figure 2 in main article and Figure A5). Those with doctor's diagnosed asthma but without current asthma (n = 429) were excluded from the analysis.

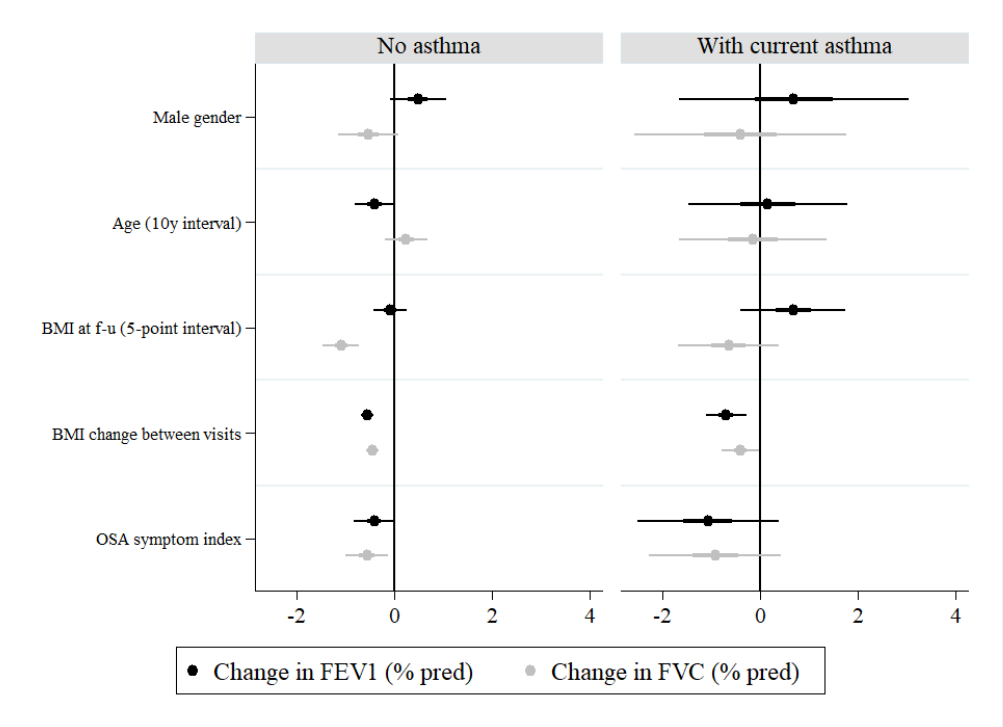

Figure A5. Multivariate mixed regression model on change in percent predicted of FEV1 and FVC between visits, showing coefficients with 50 and 95% confidence intervals. Adjusted for pack-years, baseline spirometry value, and centre. Subgraphs by current asthma. Note that variables differ in scale (*categorical:* gender; *ordinal:* OSA symptom index; *continuous:* BMI, age), affecting the interpretation of each coefficient. OSA: Obstructive sleep apnea; f-u: follow-up; % pred: percent predicted.
